# Fatale neurologische Nebenwirkung einer Anti-CD20-Antikörper-Therapie

**DOI:** 10.1007/s00108-022-01431-8

**Published:** 2022-11-23

**Authors:** Kathie Schmidt, Romy Skusa, Annette Großmann

**Affiliations:** 1 Zentrum für Innere Medizin, Medizinische Klinik III, Klinik für Hämatologie, Onkologie und Palliativmedizin, Universitätsmedizin Rostock, Ernst-Heydemann-Str. 6, 18057 Rostock, Deutschland; 2 Institut für Medizinische Mikrobiologie, Virologie und Hygiene, Universitätsmedizin Rostock, Rostock, Deutschland; 3 Institut für Diagnostische und Interventionelle Radiologie, Kinder- und Neuroradiologie, Abteilung Neuroradiologie, Campus Gehlsdorf, Universitätsmedizin Rostock, Rostock, Deutschland

**Keywords:** Progressive multifokale Leukenzephalopathie, PML, JC-Virus, Rituximab, Lymphom, Progressive multifocal leukoencephalopathy, PML, JC virus, Rituximab, Lymphoma

## Abstract

Eine 59-jährige Patientin entwickelte neurologische Defizite nach vorheriger rituximabhaltiger Lymphomtherapie. Die Konstellation aus Marklagerläsionen ohne Schrankenstörung in Groß- und Kleinhirn und DNA-Nachweis des John Cunningham Virus (JCV) aus Liquor führte zur Diagnose einer progressiven multifokalen Enzephalopathie (PML). Die PML ist eine seltene, aber tödliche Infektion des zentralen Nervensystems hervorgerufen durch eine Reaktivierung des JC-Virus, die nicht nur bei HIV-Infektionen, sondern auch iatrogen nach Einsatz monoklonaler Antikörper auftreten kann.

## Abstract (en)

Progressive multifocal leukoencephalopathy (PML) is a rare, often fatal infection of the central nervous system caused by reactivation of John Cunningham virus (JCV). The case of a 59-year-old woman presenting with neurological disorders after treatment of her relapsed lymphoma with rituximab, among others, is reported. Magnetic resonance imaging showed fast-growing white matter lesions of both hemisphere and cerebellar that were neither space-consuming nor enhancing contrast media. Clinical and radiological suspicion of PML was confirmed by detection of JCV-DNA in cerebrospinal fluid. The patient died from devastating neurological decline only 11 days after the diagnosis was made. Due to the wider indication of monoclonal antibodies in almost every medical specialty we must always consider iatrogenic PML in addition to classic PML associated with acquired immunodeficiency syndrome (AIDS).

## Anamnese

Die 59-jährige Patientin wurde wegen seit 3 Wochen bestehender Gang- und Standunsicherheit aufgrund von Schwindel und Visuseinschränkungen stationär aufgenommen. 7 Jahre zuvor war die Patientin an einem diffus großzelligen Non-Hodgkin-Lymphom (DLBCL; Stadium IIAE nach Ann Arbor) erkrankt. Nach 6 Kursen Immunchemotherapie bestehend aus Rituximab, Cyclophosphamid, Doxorubicin, Vincristin und Prednisolon (R-CHOP) gefolgt von sechs Gaben Rituximab war das DLBCL in kompletter Remission. Im Vorjahr wurde ein Rezidiv in der histopathologischen Aufarbeitung eines stenosierenden Dünndarmtumors diagnostiziert. Im Anschluss erhielt die Patientin sechs Zyklen Rituximab in Kombination mit Cyclophosphamid, Doxorubicin und Prednisolon (R-CHP) und erneut zwei zusätzliche Gaben Rituximab. Abgesehen von einer vincristinassoziierten Polyneuropathie wurde diese Immunchemotherapie bis zur jetzigen Aufnahme gut vertragen. Insbesondere Nebenwirkungen wie Infektionen waren nicht aufgetreten.

## Untersuchungsbefunde

### Klinische Untersuchung.

Patientin in leicht reduziertem Allgemeinzustand (ECOG-PS 0–1), zu allen Qualitäten orientiert. Keine B‑Symptomatik. Monokelhämatom rechts. *Neurologie: *Pupillen rund, isokor, direkt und indirekt lichtreagibel. Konvergenzreaktion intakt. Gesichtsfeld fingerperimetrisch intakt. Kein Meningismus. Kein Kalottenklopfschmerz. Keine latenten oder manifesten Paresen. Fallneigung nach rechts. *Cor, Pulmo und Abdomen *ohne pathologischen Befund. Keine palpable Lymphknotenschwellung.

### Ophthalmologie.

Gesichtsfelduntersuchung bei schlechter Compliance nicht möglich. Augeninnendruck mit 14 mm Hg im Normbereich. Kein Anhalt für ein okuläres Lymphom.

### CT Kopf nativ vom 03.01.2021.

**Figure 1:**
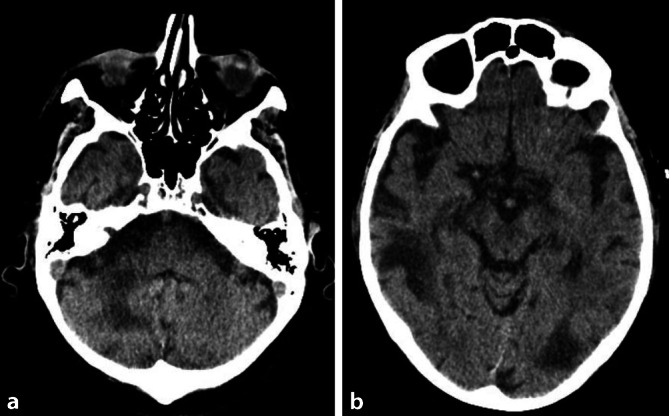

Kein Nachweis einer intrakraniellen Blutung oder eines territorialen Infarkts. Kein Liquoraufstau. Subkortikale hypodense Läsionen supra- und infratentoriell (links okzipital, rechts temporal und rechts zerebellär; Abb. 1).

### MRT Kopf mit KM vom 11.01.2021.

**Figure 2:**
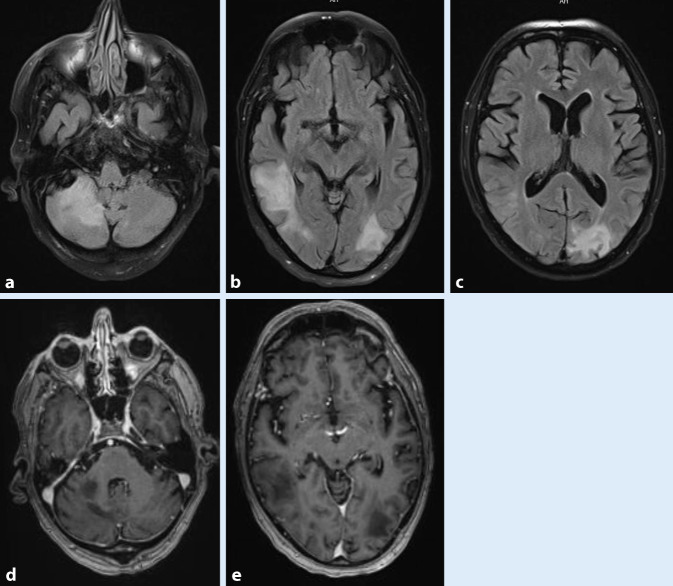

Größenprogrediente Marklagerläsionen links okzipital, rechts temporal und rechts hochparietal sowie rechts zerebellär ohne raumfordernde Wirkung oder KM-Aufnahme. Der Kortex wird von den Läsionen, die in den U‑Fasern liegen, ausgespart. Kein meningeales Enhancement (Abb. 2).

### Liquorpunktion vom 12.01.2021.

JC-Virus-DNA positiv (PCR-Verfahren). Leukozytenzahl und Laktat in der Norm (2 Mpt/l [< 5 Mpt/l] und 1,9 mmol/l [1,7–2,6 mmol/l]). Gesamtprotein erhöht (711 mg/l [150–450 mg/l]). Vereinzelt aktivierte Lymphozyten. Kein belastbarer Hinweis auf Meningeosis.

## Diagnose

- Vor dem Hintergrund der Rituximabgabe wurde anhand der Trias aus neurologischen Ausfällen, radiologischen Veränderungen und dem Nachweis von JC-Virus im Liquor die Diagnose einer progressiven multifokalen Leukenzephalopathie (PML) gestellt.

## Therapie und Verlauf

Angesichts der infausten Prognose wurde mit der Patientin ein Verzicht auf lebenserhaltende oder intensivmedizinische Maßnahmen besprochen. Der Allgemeinzustand verschlechterte sich rasch. Die Patientin war zunehmend desorientiert, verlangsamt und nahm keine Nahrung oder Medikamente mehr zu sich. Sie verstarb nur 11 Tage nach Diagnosestellung der PML.

## Diskussion

Die PML ist eine seltene, aber überwiegend tödlich verlaufende Infektion des zentralen Nervensystems, die durch eine Reaktivierung des JC-Virus (JCV) hervorgerufen wird. JCV, benannt nach dem ersten Patienten John Cunningham, gehört zur Gruppe der Polyomaviren, die 1971 durch Padgett in *Lancet* erstbeschrieben wurden [10]. Die subklinische Primärinfektion erfolgt in der Kindheit auf oralem oder respiratorischem Infektionsweg [3]. Das Virus breitet sich durch hämatogene Streuung aus, wobei Niere und zentrales Nervensystem (ZNS) als wesentliche Lokalisationen einer Viruspersistenz beschrieben werden [5]. Es persistiert üblicherweise lebenslang asymptomatisch, kann aber bei schwerer zellulärer Immunschwäche reaktiviert werden. Durch signifikante genetische Veränderungen kann es Gliazellen lytisch destruieren [8], was die Diskrepanz zwischen hohem Durchseuchungsgrad und seltenem Auftreten einer PML in Ansätzen erklärt.

Noch vor Entdeckung des JCV wurden 1958 Fälle von PML in hämatologischen Krankheitsbildern beschrieben [1]. Während der AIDS-Pandemie stieg die Inzidenz dramatisch an und flaute erst nach Einführung der Kombinationstherapie ab. Obwohl die meisten PML-Fälle AIDS-assoziiert sind, steigt seit Jahren die Inzidenz der iatrogen hervorgerufenen PML an. Grund ist die Zunahme immunmodulierender Therapien in diversen Fachrichtungen, z. B. der Anti-CD20-Therapie bei hämatologischen Erkrankungen (weitere Medikamente s. Tab. 1). Die ersten beiden Fallberichte einer PML in Verbindung mit Rituximab, einem monoklonalen CD20-Antikörper, erschienen 2008 [6]. Hierbei wirkt sich vermutlich die B‑Zell-Depletion auf den immunmodulatorischen Effekt von B‑ auf T‑Zellen aus. Typisch sind T2-hyperintense, T1-hypointense Läsionen in der Magnetresonanztomographie (MRT), die asymmetrisch über Groß- und Kleinhirn verteilt sind und konfluieren können. Betroffen sind die weiße Substanz und die U‑Fasern. Um die Hauptläsionen können kleine T2-hyperintense Läsionen gruppiert sein, was als „milky way sign“ bezeichnet wird. Obwohl es sich um einen entzündlichen Prozess handelt, reichern die Läsionen typischerweise kein Kontrastmittel (KM) an und sind nicht raumfordernd. Im Stadium der Immunrekonstitution können die Läsionen jedoch KM-affin und raumfordernd sein.

**Table 1:** 

Medikament	Indikation
Belimumab	SLE
Brentuximab-Vedotin	Hodgkin-Lymphom
Ciclosporin	Transplantation
Fingolimod	RRMS
Ibrutinib	Mantelzelllymphom, CLL, M. Waldenström
Mycophenolatmofetil	Transplantation
Natalizumab	RRMS
Obinutuzumab	CLL
Ofatumumab	CLL
Rituximab	CLL, Non-Hodgkin-Lymphom, RA, MPA
Ruxolitinib	Myelofibrose
Sirolimus	Transplantation
Tacrolimus	Transplantation
Vedolizumab	Colitis ulcerosa, Morbus Crohn

*SLE* systemischer Lupus erythematodes, *RRMS* „relapsing remitting multiple sclerosis“, *CLL* chronische lymphatische Leukämie, *RA* rheumatoide Arthritis, *MPA* mikroskopische Polyangiitis

Die Diagnose einer PML erfolgt entweder anhand des neuropathologischen Nachweises aus einer Hirnbiopsie oder aus einer Kombination von klinischen, radiologischen und mikrobiologischen Befunden (s. Tab. 2; [2]). Die neurologischen Ausfälle sind mannigfaltig und abhängig vom befallenen Hirnareal. Der laborchemische Liquorbefund ist wie in unserem Fall meist unauffällig mit allenfalls mäßiger Pleozytose und leicht erhöhtem Gesamtprotein. Der Nukleinsäurenachweis stellt das Standardverfahren zum direkten Polyomavirusnachweis dar. Aufgrund der hohen Durchseuchung ist die virusspezifische IgM- und IgG-Antikörper-Bestimmung im Serum diagnostisch nicht sinnvoll. Der Virusnachweis sollte primär aus dem Liquor erfolgen. Fällt der Befund trotz ausreichender Sensitivität des Labors negativ aus, sollte eine Hirnbiopsie erfolgen [9].

**Table 2:** 

Diagnosepfad	Diagnostische Kriterien	Diagnose							
		Definitive PML	Wahrscheinliche PML		Mögliche PML		Keine PML		
Kombination klinisch, radiologisch, laborchemisch	Laborchemisch: JCV-DNA-Nachweis in Liquor via PCR	x	x	x	x	–	–	–	–
	Klinisch: progrediente neurologische Symptome	x	x	–	–	x	–	x	–
	Radiologisch: typische MR-Veränderungen	x	–	x	–	x	–	–	x

*PML* Progressiv multifokale Leukenzephalopathie, *JCV* John Cunningham Virus

Bislang existiert keine wirksame antivirale Therapie gegen eine JCV-Infektion oder PML, was die schlechte Prognose erklärt (höchste Mortalität bei rituximabassoziierter PML mit 90 % innerhalb von 2 Monaten). Das Überleben hängt einzig von der zeitnahen Beendigung der Immunsuppression ab. Experimentelle Studien mit Checkpointinhibitoren, die die immunologische Eigentoleranz modifizieren und in einer Senkung der Viruslast münden, sind für Patienten ohne anhaltende Immunsuppression und im initialen Krankheitsstadium vielversprechend [4]. Ein innovatives Therapiekonzept beruht auf dem Einsatz von BK-Polyomavirus-spezifischen T‑Zellen in spezialisierten neurologischen Zentren. Die T‑Zellen richten sich aufgrund der Ähnlichkeit zwischen BK- und JC-Epitopen auch erfolgreich gegen das JCV [7].

Der Fall zeigt, dass bei neuen neurologischen Symptomen nach vorangegangener monoklonaler Antikörpertherapie immer eine PML differenzialdiagnostisch bedacht werden sollte. Als federführende diagnostische Methoden dienen die MRT und der DNA-Nachweis aus dem Liquor. Das Überleben hängt momentan maßgeblich von einer Rekonstitution des Immunsystems ab, wenngleich die Prognose für die Mehrzahl der Betroffenen infaust ist.

## Fazit für die Praxis

- Die progressiv multifokale Leukenzephalopathie (PML) wird durch eine Reaktivierung des John-Cunningham-Virus (JCV) bei lang andauernder Immunsuppression hervorgerufen und destruiert das ZNS mit meist tödlichem Ausgang.
- Der Anteil an iatrogenen PML durch immunmodulierende Therapien steigt.
- Die Diagnose wird aus Klinik, direktem Virusnachweis im Liquor und typischen MR-Befunden gestellt. Bei Unklarheit kann eine Hirnbiopsie forciert werden.
- Abgesehen von einer Rekonstitution des Immunsystems existiert momentan keine spezifische antivirale Therapie.
- Ein vielversprechender experimenteller Ansatz ist die Therapie mit BK-Polyomavirus-spezifischen T‑Zellen in spezialisierten Zentren.
